# Safety comparisons among monoamine oxidase inhibitors against Parkinson’s disease using FDA adverse event reporting system

**DOI:** 10.1038/s41598-023-44142-2

**Published:** 2023-11-06

**Authors:** Hiroto Asano, Yu-Shi Tian, Asuka Hatabu, Tatsuya Takagi, Mikiko Ueda, Kenji Ikeda

**Affiliations:** Graduate School of Pharmaceutical Sciences, Osaka University, 1-6 Yamadaoka, Suita, Osaka 565-0871 Japan

**Keywords:** Statistical methods, Parkinson's disease

## Abstract

Monoamine oxidase B (MAO-B) inhibitors are used to control Parkinson’s disease (PD). Selegiline, rasagiline, and safinamide are widely used as MAO-B inhibitors worldwide. Although these drugs inhibit MAO-B, there are pharmacological and chemical differences, such as the inhibitory activity, the non-dopaminergic properties in safinamide, and the amphetamine-like structure in selegiline. MAO-B inhibitors may differ in adverse events (AEs). However, differences in actual practical clinics are not fully investigated. A retrospective study was conducted using FAERS, the largest database of spontaneous adverse events. AE signals for MAO-B inhibitors, including selegiline, rasagiline, and safinamide, were detected using the reporting odds ratio method and compared. Hypocomplementemia, hepatic cyst, hepatic function abnormal, liver disorder and cholangitis were detected for selegiline as drug-specific signals. The amphetamine effect was not confirmed for any of the three MAO-B inhibitors. The tyramine reaction was detected as an AE signal only for rasagiline. Moreover, the REM sleep behavior disorder was not detected as an AE signal for safinamide, suggesting that non-dopaminergic effects might be beneficial. Considering the differences in AEs for MAO-B inhibitors will assist with the appropriate PD medication.

## Introduction

Parkinson’s disease (PD) is a highlighted neurodegenerative disorder and has become one of the most serious health problems. Based on the Global Burden of Disease 2019, a significant 155.50% global increase in the occurrence of PD has been estimated since 1990, and the prevalence reached over 8.5 million individuals. This upward prevalence trend demonstrated consistency from 1990 to 2019^1^. During the clinical progression of PD, motor symptoms, including tremors, rigidity, akinesia, imbalance, and numerous non-motor complications occur. These symptoms predominantly result from altered and abnormal neurotransmission related to deficits of predominant biogenic amines. Dopamine is the most responsible neurotransmission amine, and its relative amount in the midbrain nigrostriatal area of patients with PD decreases due to reduced or dropped out dopaminergic neurons. So far, the principal treatment of PD is to directly supplement dopamine, such as using the combination of carbidopa and levodopa or dopamine agonists, or to achieve therapeutic maintenance of dopamine by reducing the key metabolism, such as targeting catechol-O-methyltransferase or monoamine oxidase B (MAO-B) using inhibitors^2^.

MAO is an enzyme with two isoforms of MAO-A and -B, which are high-expressing in the brain and gut. Selective inhibition of MAO-B in the striatum can increase dopamine levels and show anti-PD action. However, the non-selective inhibition in the brain and gut may cause serious hypertension when taking cheese together, termed tyramine reaction. Therefore, inhibitory selectivity is important for MAO-B inhibitor development. To date, three MAO-B inhibitors, selegiline, rasagiline, and safinamide, have been approved for clinical use. These inhibitors can be used as monotherapy or add-on drugs to levodopa, improve the wear-off, delay the onset of levodopa, and prolong the mean levodopa action duration^3^.

Selegiline, an irreversible inhibitor, was approved for PD by the FDA in 1989 and has been widely used as a first-generation MAO-B inhibitor. The selectivity of MAO-B over MAO-A in the brain at low doses of selegiline has been confirmed. However, it was also reported that when administered at high doses, the selegiline level increased in plasma, indicating lower selectivities^4^. The chemical structure of selegiline contains an amphetamine skeleton, and by first-pass metabolism, selegiline can be transformed into L-amphetamine and L-methamphetamine. Despite these aspects of selegiline, which raise the possibility of adverse effects, thus far, selegiline has generally been considered a well-tolerated drug^5^, and its major adverse events are reported as headaches, dizziness, insomnia, nausea, xerostomia, and constipation^6^. Furthermore, according to meta-analysis, there is no difference in adverse event frequency between selegiline and placebo groups^7^. However, whether selegiline metabolites can cause amphetamine-like adverse events, including cardiovascular and central neural system adverse events, remains conversational. Some studies reported that cardiovascular adverse events in selegiline users might be related to amphetamine^8–13^, whereas other studies have suggested no association under ordinary use^14, 15^. Moreover, in high-dose use, whether tyramine reaction occurs remains unclear.

Rasagiline is another irreversible, selective MAO-B inhibitor^16^, which FDA approved in 2006. As rasagiline does not have an amphetamine skeleton and higher selectivities of MAO-B, rasagiline has been considered to have fewer adverse events than selegiline. Sleep disturbances have been reported to be improved by switching from selegiline to rasagiline^17^. Tyramine dietary restriction was removed based on the results from clinical studies^18^. Conversely, the package insert of selegiline still warns of the tyramine response^19^. It is important to clarify the frequency and risk of tyramine reactions using pharmacovigilance data.

Safinamide, an alpha-aminoamide derivative, has recently been on the market as the newest MAO-B inhibitor. Unlike selegiline and rasagiline, safinamide is a reversible inhibitor, and its MAO-B selectivity is much higher than the other two drugs, which may link to higher safety profiles. Furthermore, in addition to dopaminergic properties, safinamide has non-dopaminergic properties, such as inhibiting voltage-gated sodium and calcium channels and glutamate release^20, 21^. These characteristics may also distinguish safinamide from selegiline and rasagiline from the view of drug safety.

These MAO-B inhibitors play important roles in the current PD treatments. However, these drugs differ in selectivity, chemical structures, and dopaminergic properties, which may affect drug safety. Although clinical studies and meta-analyses have focused on their safety, further pharmacovigilance analyses from real-world patient data can provide deeper comprehension and address rare but important safety concerns. Therefore, this study applied AE signal detection^22^ to the data from the FDA Adverse Event Reporting System (FAERS)^23^, compared the AE signals associated with selegiline, rasagiline, and safinamide, and provided information on drug safety, particularly on the considered differences.

## Results

### Baseline information

This study involved 3784, 10,584, and 1007 records reported in FAERS (2004Q1-2022Q2) for selegiline, rasagiline, and safinamide, respectively (Table 1). The mean ages of these reports were 68, 70, and 71, and the female rates were 40–44%, suggesting no obvious differences among MAO-B inhibitors. For each drug, males were more frequently reported than females. The highest reporting proportion was confirmed in Northern America for all three MAO-B inhibitors. However, differences were observed in some reported areas. For example, selegiline has a higher reporting proportion than rasagiline and safinamide in Eastern Asia, especially in Japan (950 cases of 991 cases).

**Table 1:** Baseline information.

	Selegiline	Rasagiline	Safinamide
Age in years (mean ± SD (n))	67.8 ± 12.7 (2667)	70.0 ± 10.7 (7209)	71.4 ± 9.8 (713)
Number of combination drugs^a^ (mean ± SD)	8.5 ± 6.2	7.5 ± 5.6	7.8 ± 4.9
Monotherapy^b^ (n, %)	34 (0.8%)	883 (8.3%)	57 (5.6%)
Female (n, %)	1517 (42.6%)	3998 (40.5%)	415 (44.3%)
Area (n, %)			
Northern America	1523 (45.2%)	6082 (59.6%)	377 (40.4%)
Western Europe	201 (6.0%)	1497 (14.7%)	251 (26.9%)
Eastern Asia	991 (29.4%)	708 (6.9%)	29 (3.1%)
Northern Europe	310 (9.2%)	799 (7.8%)	62 (6.6%)
Southern Europe	231 (6.9%)	439 (4.3%)	197 (21.1%)
Eastern Europe	23 (0.7%)	169 (1.7%)	0 (0.0%)
South America	42 (1.2%)	94 (0.9%)	16 (1.7%)
Western Asia	4 (0.1%)	130 (1.3%)	0 (0.0%)
Central America	8 (0.2%)	110 (1.1%)	0 (0.0%)
Australia and New Zealand	22 (0.7%)	90 (0.9%)	1 (0.1%)
Southern Asia	5 (0.1%)	40 (0.4%)	1 (0.1%)
South-eastern Asia	11 (0.3%)	19 (0.2%)	0 (0.0%)
Southern Africa	0 (0.0%)	19 (0.2%)	0 (0.0%)
Caribbean	0 (0.0%)	5 (0.0%)	0 (0.0%)
Northern Africa	0 (0.0%)	1 (0.0%)	0 (0.0%)
Micronesia	0 (0.0%)	1 (0.0%)	0 (0.0%)

In this table, unreported data were excluded from the calculation of both the count (n) and the percentage (%).

^a^The number of combination drugs was counted when other drugs were reported for the same primary ID.

^b^Monotherapy is counted when no other drugs are reported for one primary ID.

### AE signals detected for MAO-B inhibitors

The AE signals detected for selegiline, rasagiline, and safinamide were 585, 672, and 254 PTs (Tables S4–S7). Common signals in all drugs were 111 PTs and were tabulated to primary SOCs for a concise view (Table 2). At the SOC level, signals included in “nervous system disorders” (n = 30, 27.0%), “psychiatric disorders” (n = 27, 24.3%), and “injury, poisoning, and procedural complications” (n = 18, 16.2%) were frequently detected, counting for more than 65%. Parkinsonism-like signals, such as on–off phenomena (selegiline OR 37, 95% CI 29–46; rasagiline OR 45, 95% CI 40–51; safinamide OR 78, 95% CI 58–104), are commonly detected in neurological disorders. Regarding mental disorders, impulse control disorders (selegiline OR 92, 95% CI 71–120; rasagiline OR 72, 95% CI 60–87; safinamide OR 12, 95% CI 3–46), hypersexuality (selegiline OR 145, 95% CI 115–184; rasagiline OR 100, 95% CI 84–120; safinamide OR 73, 95% CI 39–136), and others were commonly detected. As for injury, poisoning and procedural complications, in addition to falls, fractures, and injuries-related signals associated with motor function, eight stoma-related signals were detected. These might have occurred due to enteral therapy for PD.

**Table 2:** Common Signals (SOC level) and the number of preferred terms (PT).

SOC	Number of PT
Nervous system disorders	30 (27.0%)
Psychiatric disorders	27 (24.3%)
Injury, poisoning and procedural complications	18 (16.2%)
General disorders and administration site conditions	10 (9.0%)
Product issues	7 (6.3%)
Respiratory, thoracic and mediastinal disorders	3 (2.7%)
Musculoskeletal and connective tissue disorders	3 (2.7%)
Gastrointestinal disorders	3 (2.7%)
Surgical and medical procedures	2 (1.8%)
Vascular disorders	1 (0.9%)
Social circumstances	1 (0.9%)
Skin and subcutaneous tissue disorders	1 (0.9%)
Renal and urinary disorders	1 (0.9%)
Metabolism and nutrition disorders	1 (0.9%)
Investigations	1 (0.9%)
Infections and infestations	1 (0.9%)
Eye disorders	1 (0.9%)
Total	101

Alternatively, 308, 367, and 87 PTs were detected as drug-specific signals for selegiline, rasagiline, and safinamide. The SOCs of these PTs are shown in Table 3. SOC of “hepatobiliary disorders” contained only selegiline-specific signals. Meanwhile, “neoplasms benign, malignant and unspecified” and “vascular disorders” predominantly contained rasagiline-specific signals. No SOC only contained safinamide-specific signals. However, the number of safinamide-specific signals for eye disorders was higher than that of the other two drug-specific signals.

**Table 3:** Product-specific signals (SOC level) and the number of preferred terms (PT).

SOC	Selegiline	Rasagiline	Safinamide
Investigations	37 (12.0%)	22 (6.0%)	6 (6.9%)
Nervous system disorders	35 (11.4%)	49 (13.4%)	16 (18.4%)
Cardiac disorders	32 (10.4%)	7 (1.9%)	2 (2.3%)
Injury, poisoning and procedural complications	30 (9.7%)	43 (11.7%)	10 (11.5%)
Gastrointestinal disorders	23 (7.5%)	21 (5.7%)	0 (0.0%)
Psychiatric disorders	22 (7.1%)	52 (14.2%)	3 (3.4%)
Respiratory, thoracic and mediastinal disorders	16 (5.2%)	12 (3.3%)	6 (6.9%)
Infections and infestations	14 (4.5%)	12 (3.3%)	7 (8.0%)
General disorders and administration site conditions	11 (3.6%)	42 (11.4%)	6 (6.9%)
Surgical and medical procedures	11 (3.6%)	12 (3.3%)	4 (4.6%)
Musculoskeletal and connective tissue disorders	9 (2.9%)	6 (1.6%)	3 (3.4%)
Social circumstances	8 (2.6%)	5 (1.4%)	2 (2.3%)
Neoplasms benign, malignant and unspecified (incl cysts and polyps)	7 (2.3%)	24 (6.5%)	1 (1.1%)
renal and urinary disorders	7 (2.3%)	5 (1.4%)	2 (2.3%)
Product issues	6 (1.9%)	10 (2.7%)	1 (1.1%)
Skin and subcutaneous tissue disorders	6 (1.9%)	8 (2.2%)	2 (2.3%)
Metabolism and nutrition disorders	6 (1.9%)	7 (1.9%)	2 (2.3%)
Eye disorders	5 (1.6%)	4 (1.1%)	8 (9.2%)
Reproductive system and breast disorders	5 (1.6%)	4 (1.1%)	0 (0.0%)
Blood and lymphatic system disorders	5 (1.6%)	3 (0.8%)	1 (1.1%)
Hepatobiliary disorders	5 (1.6%)	0 (0.0%)	0 (0.0%)
Vascular disorders	2 (0.6%)	12 (3.3%)	3 (3.4%)
Congenital, familial and genetic disorders	2 (0.6%)	2 (0.5%)	0 (0.0%)
Endocrine disorders	2 (0.6%)	1 (0.3%)	1 (1.1%)
Immune system disorders	1 (0.3%)	2 (0.5%)	0 (0.0%)
Pregnancy, puerperium and perinatal conditions	1 (0.3%)	1 (0.3%)	0 (0.0%)
Ear and labyrinth disorders	0 (0.0%)	1 (0.3%)	1 (1.1%)
Total	308	367	87

### Confirmation of tyramine reaction

The tyramine reaction was detected as AE signals only for rasagiline (OR 626, 95% CI 192–2038) and not for selegiline or safinamide. However, four cases reported tyramine reactions when using Emsam, a patch formulation of selegiline, as an antidepressant (Table S8).

### Non-dopaminergic effects of safinamide

We further focused on whether “REM sleep behavioral disorder” (RBD) was detected as a signal in selegiline, rasagiline, and safinamide as an AE signal. As expected, this AE signal was detected only for selegiline (OR 23, 95% CI 7–70) and rasagiline (OR 51, 95% CI 31–82), other than safinamide, suggesting that non-dopaminergic effects suppress the appearance of RBD. Additionally, using data from 2015Q1, when safinamide was approved, signal detection was also performed to validate the robustness of the result. RBD was also detected in selegiline and rasagiline but not in safinamide (Tables S9–11).

### Confirmation of amphetamine effects

Dice and Simpson’s coefficients of AE signals between amphetamine and MAO-B inhibitors are shown in Table 4. The similarities were no more than 0.61 for all comparisons, suggesting that none of the MAO-B inhibitors strongly share the AE signals with amphetamine. Moreover, no obvious difference was detected using either coefficient when comparing the similarities among the three MAO-B inhibitors. Sensitivity analyses showed that this conclusion was robust.

**Table 4:** The Dice’s and Simpson’s coefficients of AE signals were detected between amphetamine and each MAO-B inhibitor.

	Coefficients	Amfetamine	Selegiline	Rasagiline	Safinamide
Total signals		1530	585	672	254
All signals	D^a^	Ref.	0.18	0.23	0.12
	S^b^	Ref.	0.33	0.37	0.43
Top 50% in odds ratio	D	Ref.	0.10	0.11	0.05
	S	Ref.	0.11	0.11	0.10
Top 25% in odds ratio	D	Ref.	0.05	0.06	0.03
	S	Ref.	0.07	0.08	0.04
Top 10% in odds ratio	D	Ref.	0.02	0.02	0.00
	S	Ref.	0.05	0.05	0.01
PTs included in SOCs (psychiatric, neurological, cardiac, and vascular disorders)	D	Ref.	0.36	0.43	0.22
	S	Ref.	0.48	0.54	0.60
PTs included in SOCs (psychiatric, neurological)	D	Ref.	0.40	0.47	0.23
	S	Ref.	0.58	0.59	0.61

^a^D, Dice’s coefficient; ^b^S, Simpson’s coefficient.

Next, the overlapping extent of warnings listed in the package insert of amphetamine^24^ and AE signals detected for MAO-B inhibitors was confirmed (Table 5). No obvious difference was detected between selegiline and the other MAO-B inhibitors.

**Table 5:** The headlines and subheadlines listed as WARNINGS in an amphetamine package insert and the number of the corresponding MedDRA terms in each product signal.

Headline	Subheadline	The number of corresponding MedDRA terms (PT)	Selegiline	Rasagiline	Safinamide
Serious cardiovascular events	Sudden deaths	3	1	0	0
	Stroke	11	0	2	0
	Myocardial infarction	41	1	2	1
	Hypertension	90	8	8	2
Psychiatric adverse events	Pre-existing psychosis worse	1	1	1	1
	Bipolar illness & Emergence of new psychotic or manic symptoms	7	2	2	1
	Aggression	1	1	1	1
Long-term suppression of growth		3	0	0	0
Seizures		91	3	2	3
Peripheral vasculopathy, including Raynaud’s phenomenon		63	0	2	2
Serotonin syndrome		1	1	1	1
Visual disturbance		96	2	4	5

## Discussion

This study focused on three anti-PD MAO-B inhibitors: selegiline, rasagiline, and safinamide. We used the AE signal detection method by mining the FAERS database to confirm potential AE. Previous studies reported that the incidence rate of PD increases rapidly after 60, that of males is higher than females, and there are also race/ethnicity differences^25^. Although the AE reports in FAERS cannot be directly considered as the incidence of PD, similar trends were confirmed in this study. Patients with PD who experienced adverse events related to MAO-B inhibitors had an average of approximately eight drugs concurrently. A previous study indicated that the mean number of drugs used by PD patients was 3.8. The mean number for those aged 70 to 79 was 4.7 drugs^26^. Our results exceeded the numbers reported in this previous report. Nonetheless, the findings of multiple drug usage among PD patients remain consistent, likely due to the typical characteristics of PD, which are often accompanied by both physical and mental comorbidities^27^. We also found reporting frequency differences of these drugs among the reporting countries. One reason for these regional differences may be associated with different approval years. Selegiline was the only approved MAO-B inhibitor in Japan from 2007 until 2018. Rasagiline and safinamide were approved later than selegiline in Europe (2005 and 2015), the USA (2006 and 2017), and Japan (2018 and 2019). This may explain why selegiline had a higher reporting frequency in Eastern Asia, especially Japan.

Drug-specific and common PT signals were detected in this study. However, as PT terms are considered too detailed to understand, we gathered PT signals and assigned them to corresponding SOC terms for a concise discussion.

SOC of “hepatobiliary disorders” contained only selegiline-specific PTs. We further separated the reporting data and compared the signals from the Japanese and other regional reports. Four PT signals in this SOC were detected in Japanese reports, but only one PT signal in other regions (Table S12). Furthermore, the Japanese reported the majority of these PTs. For example, the hepatic cyst was reported ten times in the whole data, and eight times were reported from Japan (Table S12). It has been reported that MAO-A is related to the pathogenesis of liver diseases via serotonin^28^. Although selegiline is considered an MAO-B selective inhibitor, a high blood concentration of selegiline could cause MAO-A inhibition due to decreased MAO-B selectivity^4^. Selegiline is metabolized by CYP2C19 or CYP2B6^29, 30^ enzyme polymorphisms, interaction with other drugs, and other potential factors may elevate its blood concentration. It is known that approximately 5% and 20% of CYP2C19 Poor Metabolizers (PM) are in the Caucasian race and Japanese^31^. Moreover, in a clinical trial of selegiline, 2 of 100 adverse events that occurred in Japanese patients were hepatobiliary diseases (gallstones and acute cholecystitis / obstructive jaundice, pancreatic duct obstruction, and pancreatic cancer)^32^. The above information might suggest the possibility of selegiline-specific hepatobiliary-related events and a higher frequency in Japanese patients. However, FAERS issues exist, including reporting bias^33^ and lack of patient details. We cannot clarify whether selegiline is more relevant to liver/gallbladder diseases than the other two MAO-B inhibitors and exhibits a higher frequency in Japanese and leave this observation as a hypothesis. Further validation is needed. Nevertheless, attention to hepatobiliary disorders should be paid.

PTs included in SOCs of “vascular disorders” and “neoplasms benign, malignant and unspecified” are especially detected in rasagiline. Several clinical trials have reported an association between rasagiline and blood pressure regarding vascular disorders. The TEMPO Study, a clinical trial conducted in patients with early PD, reported a significant increase in supine systolic blood pressure versus placebo in the 2 mg group^34^. Stern et al. reported that patients with early PD slightly tended to decrease blood pressure at 2 and 4 mg/day of rasagiline^35^. According to the PRESTO Study, rasagiline 0.5 mg/day decreases systolic and diastolic blood pressures in patients with advanced PD while standing^36^. The LARGOR study reported a 2% incidence of postural hypotension at 1 mg/day of rasagiline^37^. The results from these clinical studies have shown that rasagiline may affect vascular disorders, especially blood pressure, suggesting generally in line with our findings.

Regarding neoplasms, the TEMPO Study reported that 3 of 132 patients in the 2 mg dose group were newly diagnosed with malignancies (malignant melanoma, prostate cancer, squamous cell carcinoma)^34^. In the second phase of the TEMPO study, 5 of 371 patients were newly diagnosed with malignancies (colon cancer, two cases of squamous cell carcinomas of the skin, basal cell carcinoma, and melanoma)^38^. Additionally, the ADAGIO study reported that one patient who took rasagiline 1 mg/day had melanoma at week 72^39^. Regarding the relationship between tumors and PD, it was reported that PD correlates positively with melanoma and negatively with other tumors^40^. Recently, rasagiline was reported to be a potential melanoma risk factor^41^. In this study, melanoma-associated signals were detected only in rasagiline, supporting the possibility that rasagiline may be a risk factor for melanoma. Although the influence of other risk factors such as lifestyle, underlying disease, and database factors such as reporting bias should continue to be investigated, attention should be paid to tumor-related adverse events, especially melanoma, when using rasagiline.

Signals associated with eye disorders were more for safinamide. Animal experiments using rats showed drug-induced retinal atrophy, suggesting that an ophthalmological examination for humans is needed before using safinamide. In phase 3 clinical trials (015, 017, and 018), blurred vision or cataract was also reported as an AE of safinamide^42^. Despite the small number of reports and lack of background factors, ocular disturbances with safinamide should be noted.

In a clinical study of tyramine (50–75 mg) in 72 rasagiline-treated and 38 placebo-treated patients, there were no tyramine reactions in the rasagiline monotherapy group (n = 38). In contrast, tyramine reactions were observed in 3 of 22 patients who received 0.5 mg/day of rasagiline in the levodopa combination group^43^. In this study, tyramine reaction was detected as an AE signal of rasagiline with a low reporting frequency of n = 3 (Table S8). However, by confirming all the data since 2004, only 37 cases of tyramine reactions, a serious adverse event, were reported for all approved drugs, and rasagiline accounted for about 8%. We did not detect the tyramine reaction as a signal for selegiline. However, a previous study showed that the tyramine susceptibility coefficient (ratio of TYR30 between placebo and actual drug administration) of selegiline (5 mg, twice daily) was higher than that of rasagiline (1 mg/day)^18^. Furthermore, tyramine reactions were reported with Emsam (n = 4) (Table S8). Therefore, although the incidence rate is low, rasagiline and selegiline should be used with caution in the tyramine response.

Recently, a study focused on the glutamate release of safinamide and reported its efficacy in RBD^44^. The prevalence of RBD in PD patients is 46–58%^45^. RBD is a disease that exhibits violent movements during rapid eye movement (REM) sleep. Glutamate, glycine, and gamma-aminobutyric acid play an important role in the pathogenesis of RBD^46^. Against this background, we focused on RBD and found that it was not detected as an AE signal, only for safinamide, suggesting that the non-dopaminergic effects of safinamide are beneficial. However, we cannot exclude the possibility that RBD was not detected as an AE signal in safinamide due to a small number of reports. Ronconi et al. reported the prescription frequency of MAO-B inhibitors as 1059 patients used MAO-B inhibitors in 2017, 502 patients (47%) were selegiline, 161 patients (15%) were rasagiline, and 396 patients (37%) were safinamide in Italy^47^. Although the global prescription rate of MAO-B inhibitors is not reported, the reports for safinamide can be expected to increase. Further investigation on the RBD-protecting effects of safinamide should be continued in the future.

Amphetamine has been associated with the central nervous system (CNS) and cardiovascular toxicity^48, 49^. In amphetamine users, common symptoms have been reported, including agitation, hallucinations, suicidal behavior, and chest pain^50^. Cardio-cerebrovascular deaths have also been reported^51^. L-amphetamine, a metabolite of selegiline, has approximately 1/10 the pharmacological activity of D-amphetamine but has similar activity in inhibiting dopamine uptake in the striatum^52^. This study confirmed whether amphetamine effects associated with MAO-B inhibitors, particularly selegiline, exist from two aspects, and the amphetamine effect of selegiline is not higher than the other two drugs. This result supports that a clinical trial of selegiline has not identified any causal adverse events that can be considered amphetamine effects^32^, and studies using squirrel monkeys and rats have reported no amphetamine effects at normal doses of selegiline^14, 15^. In summary, we could not confirm a clear amphetamine effect associated with selegiline and other MAO-B inhibitors from these two comparisons.

VigiBase^53^, the foundational database underlying VigiAccess^54^, is publicly managed by the WHO and serves as a repository for adverse event reporting data collected worldwide. VigiBase integrates causal assessment into the data aggregation. Although this causal assessment may introduce selection bias when detecting potential signals, information reliability is generally higher than in spontaneous reporting databases such as FAERS. Therefore, we reaffirmed our results using VigiAccess (Tables S13–S15). As a result, no significant disparities were observed. Insights from prior research investigating the overlap of signals between VigiBase and FAERS suggest that while potential differences might arise due to regional approvals and market penetration, the signal detection outcomes between the two databases tend to be consistent^55^. Our findings in this study align with this trend, further substantiating the validity of signal detection conducted using FAERS.

Since our study was based on the FAERS, some limitations could not be excluded. FAERS is a spontaneous reporting database with several unavoidable inheritance shortcomings. For example, the expressions are not unified and include misspellings and omissions, and duplications and reporting bias exist^33^. This study attempted to reduce these limitations by curating the database before detecting signals. Misspellings and omissions were solved via the method used. For the duplication, reports with an exact match were considered duplicated and left one for each case. However, reports with unfilled information cannot be judged. Thus, the possibility of duplicate counting might still exist, which may affect the result. Additionally, the influence of combination uses exists in this study. Adverse events in FAERS are reported cumulatively for all drugs taken by a patient^56^, and there is a lack of adequate information concerning the periods of drug administration^57^. Therefore, it is unable to separate the monotherapy users from others correctly. Instead, the patient number of monotherapy was counted according to whether other drugs were reported. However, AE signals may be overlooked if we only use these patients for signal detection. Therefore, we did not conduct signal detection for monotherapy only. Furthermore, this study did not consider external factors such as the administration’s safety information, market trends, and approval timing. These factors may also affect adverse event reporting. As outlined in the Good Pharmacovigilance Practices and Pharmacoepidemiologic Assessment by the FDA, signal detection does not establish a causal relationship between a drug and an adverse event; rather, it raises awareness regarding potential new risks^58^. This limitation also exists in this study. Therefore, further investigation of AE associated with MAO-B inhibitors is still needed.

## Method

### Data source

This study used data reported in the largest spontaneous AE-reporting database, FAERS. FAERS collects reports of adverse drug events from experts, consumers, and manufacturers and publishes them quarterly in seven tables (i.e., DEMO, DRUG, INDI, OUTC, REAC, RPSR, and THER). We retrieved reports from 2004Q1 through 2022Q2 from the official FAERS website^23^.

### Preprocessing

Since FAERS is a voluntary reporting system, it contains reporting fluctuations, inconsistencies, and errors, influencing the AE reporting frequency. Therefore, before signal detection, data were preprocessed and standardized. Data in DEMO, DRUG, INDI, REAC, and THER were curated.

The DEMO table contains patient demographic information, such as age, body weight, gender, event date, and reporting country. Body weight recorded in various units (i.e., kgs, kg, gms, mg, lbs, lb) was transformed into kg, and data reported in other units were not used. Age recorded in various units was transformed into the year, and those that could not be transformed were not analyzed. Event dates formatted only as YYYY-MM-DD were used for analysis. Reporting countries were standardized per ISO3166^59^. Incorrect entries were manually corrected, and country codes were assigned.

The DRUG table reports the used drugs when an AE is reported. However, there are no strict restrictions on the drug names in FAERS. That is, generic names, brand names, ingredient names, abbreviations, and further fluctuations were recorded, which will significantly influence the accuracy of the signal calculation. Therefore, we cleaned the drug names using KEGG DRUG^60–63^ below. Firstly, we generated a dictionary of drug names with a key of KEGG ID (hereinafter KEGG DICT). Subsequently, we assigned a KEGG ID to each drug name obtained from FAERS per KEGG DICT. In the case of a combination drug, records were created for each ingredient, and KEGG IDs were assigned. The ingredient names (prod_ai) were used when drug names were not reported or could not be found in KEGG DICT. Next, the KEGG DICT was updated with the mapped drug names of FAERS. Finally, those drug names that could not be mapped earlier were rechecked using the updated KEGG DICT. Drug names that could not be mapped in the above steps were used per se. The fluctuations of selegiline, rasagiline, and safinamide were unified (Table S1).

The REAC table reports AEs per the Preferred Term (PT) in the Medical Dictionary for Regulatory Activities (MedDRA)^64^. MedDRA is a glossary of medical terms for symptoms, signs, and diseases developed by the International Council for Harmonisation of Technical Requirements for Pharmaceuticals for Human Use (ICH) to facilitate rapid and accurate international transfer of medical information. This glossary has a five-level hierarchical structure, System Organ Class (SOC), High-Level Group Term (HLGT), High-Level Term (HLT), PT, and Lowest-Level Term (LLT). Although REAC tables collected PTs as terminology of AEs, data fluctuations due to various versions of MedDRA or unstandardized inputs exist. Therefore, we updated all the used records in REAC per the latest version of MedDRA (ver 25.0).

The THER table contains medication information. Only the start and end dates described in YYYY-MM-DD format were used for analysis.

The INDI table contains the reasons for medication. Terminology is not strictly restricted to PTs. Therefore, we unified the terminology to the latest MedDRA PTs. Records that cannot be transformed were excluded from the analysis.

### Integrating tables and aggregating data

Data were integrated with primaryid and drug_seq as the keys for Drug, THER, and INDI, and primaryid for other tables. This study considered the records with the same drug name, start date, end date, indiction, AE name, gender, event date, body weight, age, and reporter_country as the same. All except the last report were deleted to deal with the duplications per FDA recommendations. In addition, we excluded reports in which the medication start date was later than the date of AE.

Demographic information, including patient age, gender, and reporting country, was aggregated. Gender reported other than female or male were excluded from the aggregation. ISO 3166 categorized reporting countries were converted to geographical regions using UN M49^65^ and tabulated. Unknown records were excluded from the aggregation. Mean and standard division (SD) were calculated for age, and proportions were calculated for gender and reporting countries.

### AE signal detection

**Figure 1:**
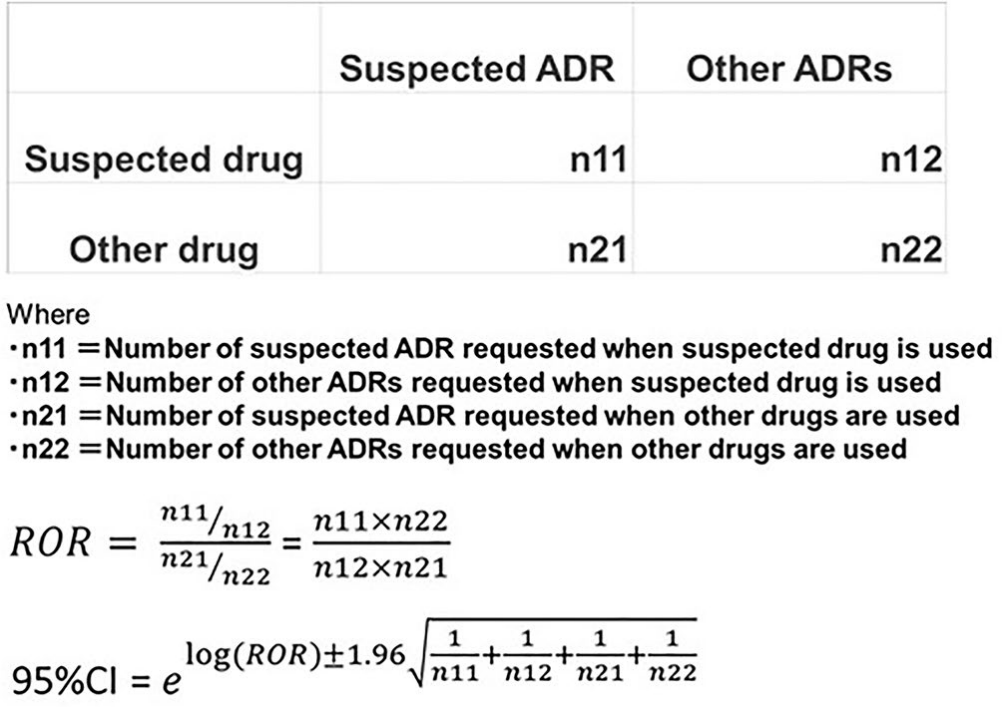
ROR method used for AE signal detection.

AE signals were detected using the reporting odds ratio (ROR) method^22^, which is used by regulatory authorities such as the Lareb in the Netherlands. When the lower limit of the 95% confidence interval of the ROR and reporting number (n_11_) are greater than 1^66^, it is defined as a signal. The odds of a suspected adverse event occurring when a particular drug is administered are higher than not (Fig. 1).

Selegiline is indicated for PD as an oral drug and depression as a patch formulation. Because this study focused on only AEs associated with anti-PD use, we considered selegiline reported in patch formulations (i.e., Emsam, parenteral, topical, transdermal, and patch) or depression-related indications (Table S2) as other drugs. For a concise view in further comparison, the detected AE signals were also tabulated to SOC per the primary SOC terminology.

### Confirmation of amphetamine effects

Amphetamine effects have been a concern for selegiline. However, the definition is unclear. This study investigated the effect associated with selegiline using the two following methods.

Firstly, the similarities between the AE signals detected for amphetamine and MAO-B inhibitors were compared using Dice’s and Simpson’s coefficients (Eqs. 1, 2). These coefficients are the methods used for similarity calculations^67^.

1 $$Dice\left( {X,Y} \right) = \frac{{2 \times \left| {X \wedge Y} \right|}}{\left| X \right| + \left| Y \right|}$$

2 $${\text{Simpson}}\left( {X,Y} \right){ } = { }\frac{{\left| {X \wedge Y} \right|}}{{\min \left( {\left| X \right|,\left| Y \right|} \right)}}$$

Here, *X* represents the set of AE signals for amphetamine, and *Y* represents that for an MAO-B inhibitor (i.e., selegiline, rasagiline, or safinamide). The greater these coefficients, the more the similarities. By comparing the similarities between the AE signals for amphetamine and each MAO-B inhibitor, the differences in amphetamine effects among selegiline, rasagiline, and safinamide can be confirmed. Moreover, because AE signals with higher odds ratios can be considered serious, sensitivity analyses using top 50%, 25%, and 10% AE signals detected for amphetamine were conducted. Furthermore, AE signals of particular concern with amphetamines (i.e., PTs belonging to neurological, psychiatric, vascular, and cardiac SOCs, or PTs belonging to the neurological and psychiatric SOCs only) were also confirmed.

Secondly, the overlapping extent of warnings listed in the package insert of amphetamine and AE signals detected for MAO-B inhibitors, particularly selegiline, was confirmed. The warnings include serious cardiovascular events, psychiatric AEs, long-term growth suppression, seizures, peripheral vasculopathy, including Raynaud’s phenomenon, serotonin syndrome, and visual disturbance. However, the terminology does not match AE signals. Thus, we used MedDRA and Standardised MedDRA Queries (SMQ) to map these WARNINGS to MedDRA terminology (Table S3). Subsequently, the overlap of these warnings and AE signals detected for MAO-B inhibitors were compared.

### Supplementary Information

Supplementary Tables.

## Data Availability

All data we use in this study is available to the following URL: https://fis.fda.gov/extensions/FPD-QDE-FAERS/FPD-QDE-FAERS.html.
